# Micro-Computed Tomography Whole-Block Imaging Reveals Origin and Path of Rectal Cancer Tumor Deposits: A Pilot Study

**DOI:** 10.3390/diagnostics14161704

**Published:** 2024-08-06

**Authors:** Canan Firat, Nil Urganci, Alexei Teplov, Emine Cesmecioglu, Nilay Bakoglu, Efsevia Vakiani, Peter Ntiamoah, Martin R. Weiser, Julio Garcia-Aguilar, Meera Hameed, Yukako Yagi, Jinru Shia

**Affiliations:** 1 Department of Pathology, Memorial Sloan Kettering Cancer Center, New York, NY 10065, USA; canan.firat@mountsinai.org (C.F.); urgancin@mskcc.org (N.U.);; 2 Department of Pathology, Marmara University Research and Education Hospital, Istanbul 34899, Turkey; 3 Department of Surgery, Memorial Sloan Kettering Cancer Center, New York, NY 10065, USA

**Keywords:** micro-CT, rectal cancer, three-dimensional images, tumor deposit, whole-block imaging

## Abstract

In colorectal carcinoma (CRC), tumor deposits (TDs) are described as macroscopic/microscopic nests/nodules in the lymph drainage area discontinuous with the primary mass, without identifiable lymph node (LN) tissue, and not confined to vascular or perineural spaces. A TD is categorized as pN1C only when no bona fide LN metastasis exists. However, there has been an ongoing debate on whether TDs should be counted as LNs. The fact that the origin of TDs is not fully understood adds further uncertainty. This pilot study aims to evaluate whether whole-block imaging by micro-computed tomography (micro-CT WBI) that enables three-dimensional reconstruction of whole-mount (WM) blocks can serve as a tool to assess the origin and path of CRC TDs. We evaluated whole-slide imaging (WSI) and micro-CT WBI of 20 WM blocks from a rectal cancer resection that contained TDs. Each TD was tracked through the contiguous blocks to define their origin and path. Of eleven TDs identified on WSI, six were detected on WBI. Strikingly, six of six TDs trackable through the blocks on WBI revealed an origin from the main tumor. This pilot study provided evidence that micro-CT WBI can serve as an effective tool to evaluate the origin and path of CRC TDs.

## 1. Introduction

Whole-block imaging using micro-computed tomography (micro-CT WBI) is a recently emerged imaging modality that applies micro-CT to formalin-fixed paraffin-embedded (FFPE) tissue blocks and allows three-dimensional (3D) reconstruction of tissue structures at a microscopic level resolution. Preliminary studies have demonstrated its ability to distinguish tumoral tissues from non-tumoral tissues [1,2]. A recent study from our group has further shown that the added information from the third dimension (not attainable from conventional 2D histology) enhances the detection of vascular invasion and allows the quantitation of the volume of lymph node metastasis, thus complementing conventional histology [3].

A “tumor deposit (TD)” is a pathologic finding of colorectal cancer resection specimens that has been associated with an unfavorable prognosis [4,5,6]. A TD refers to macroscopic or microscopic tumor nests or nodules within the pericolorectal adipose tissue in the lymph drainage area of a primary carcinoma that is discontinuous with the primary tumor. The current AJCC categorizes the presence of a TD as pN1c but exclusively for cases that do not have concurrent bona fide lymph node metastasis; when bona fide lymph node metastasis exists, the tumor deposit is no longer factored into the staging [7]. Debates are ongoing about the true biological relevance of tumor deposits and whether pN1c is the most appropriate approach to capture this relevance.

An essential aspect in determining the importance of tumor deposits relates to their origin. The prevailing theory holds that tumor deposits originate from extramural vascular invasion (EMVI) or extramural perineural invasion (EM-PNI), or they may represent completely tumor-replaced lymph nodes. As each of the three purported origins denotes aggressive tumor behavior, this origin theory provides a plausible basis for some of the recent studies that have indicated a strikingly more substantial adverse effect of tumor deposits than bona fide lymph node metastasis on patients’ clinical outcomes [8]. These origins, however, remain to be substantiated by solid evidence. There are also other related and important questions, including whether and how the adverse effect of tumor deposits differs according to their origins and whether there are tools that can detect the origins. 

On this premise, we designed this pilot study to explore the utility of micro-CT WBI in assessing TDs. We hypothesized that the spatial information obtained from the third dimension via micro-CT WBI can provide valuable data in delineating how tumor deposits form. 

## 2. Materials and Methods

### 2.1. Case Selection

The study was approved by the Institutional Review Board of Memorial Sloan Kettering Cancer Center. To obtain a side-by-side comparison between WSI and WBI, we studied a specimen obtained from an abdominoperineal resection that was performed on a rectal cancer patient at our institution. The resection specimen was processed via a whole-mount approach (WMA) as described previously [9,10]. Briefly, the steps involved were the following: inking the circumferential resection margin using different colors to distinguish anterior, posterior, right lateral, and left lateral aspects, fixing the rectal specimen with the tumor region intact/not-opened for ≥48 h, cross-sectioning at 4 mm intervals of the entire tumor region, including the entire portion distal to the tumor and up to 2 cm proximal to it, obtaining WM blocks and hematoxylin and eosin (H&E) slides of each cross-section, and digitizing both gross and microscopic pictures of WM slices and WM slides, respectively. A total of twenty formalin-fixed paraffin-embedded (FFPE) WM blocks were obtained from the study case.

### 2.2. Whole-Slide Imaging Analysis

All WM H&E glass slides were digitally scanned using a NanoZoomer S60 (Hamamatsu Photonics, Hamamatsu, Japan) at 20× magnification (0.46 m/pixel) to create virtual slides, which enabled side-by-side comparison with WBI. Tumor, mucin, TD, EMVI, and PNI were revealed by WSI.

### 2.3. Whole-Block Imaging Analysis

**Figure 1:**
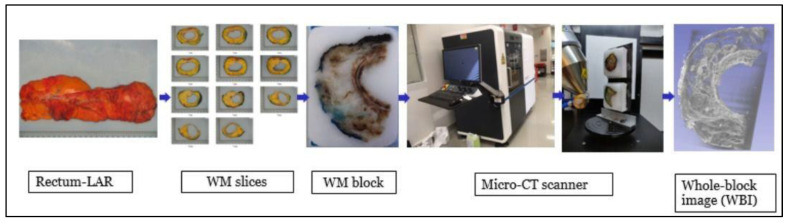
Diagram of the workflow. Whole-mount (WM) slices were obtained from a rectal cancer resection specimen. WM blocks were scanned by micro-CT scanner to generate 3-dimensional whole-block images.

The workflow is depicted in Figure 1. Each of the twenty FFPE WM blocks was scanned using a custom-made micro-CT imaging system (3D Histech Ltd., Budapest, Hungary) to obtain WBI as previously described [1,2]. Images were automatically reconstructed using modified Feldkamp-filtered back-projection algorithms with CD3Pro3D (Nikon Metrology, Leuven, Belgium) with appropriate parameters per image type and scanned resolution and digitally stained to obtain an H&E-like appearance. The reconstructed 3D model data were visualized and analyzed using VG Studio M2.2.6 (Volume Graphics GmbH, Heidelberg, Germany) and/or Dragonfly 4.2 (ORS, Montreal, QB, Canada). These applications allow the construction and analysis of 3D data, separation, and extraction of the object of interest for data to be measured, quantified, and filtered in all dimensions. The same pathological features (tumor, mucin, TD, EMVI, and EM-PNI) were evaluated using WBI.

## 3. Results

**Figure 2:**
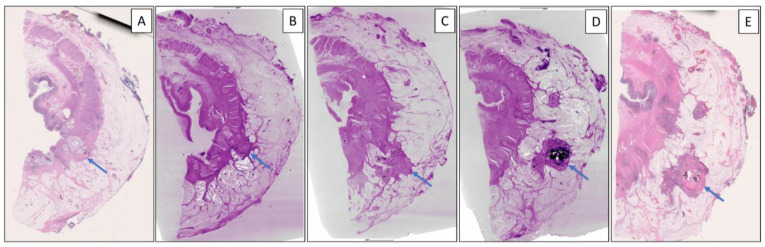
Demonstration of a tumor deposit formation by conventional H&E slide image using WSI and micro-CT WBI. (**A**) The tumor (blue arrow) slightly pushes into the adipose tissue on WSI (**B**–**D**) Micro-CT WBI of the next three consecutive whole-mount blocks demonstrates the migration of tumor cells from the primary tumor to the adipose tissue to form a tumor deposit at a resolution of 25.4–33.5 μm/voxel. (**E**) WSI of the fully formed tumor deposit (blue arrow). (Micro-CT WBI: whole-block imaging using micro-computed tomography; WSI: whole-slide imaging).

Histological assessment indicated that the study case was a poorly differentiated rectal adenocarcinoma, with no significant response to neoadjuvant therapy (tumor regression score 3, modified Ryan scheme), that was staged as ypT3N2b with eight of nineteen lymph nodes being positive. The pathologic T-stage of the tumor was accurately revealed by WSI and micro-CT WBI. Micro-CT WBI successfully detected a mucin component in the tumor that was identified on WSI. The number of LNs counted on WSI did not change with micro-CT WBI. On WSI, eleven TDs, as well as foci of EMVI and EM-PNI were identified. On micro-CT WBI, all major histological features (tumor, mucin, EMVI, LN, and TD) were identified at a resolution of 7.8–6.0× magnification (~25.4–33.5 μm/voxel) and paired with an available H&E slide image (Figure 2).

**Figure 3:**
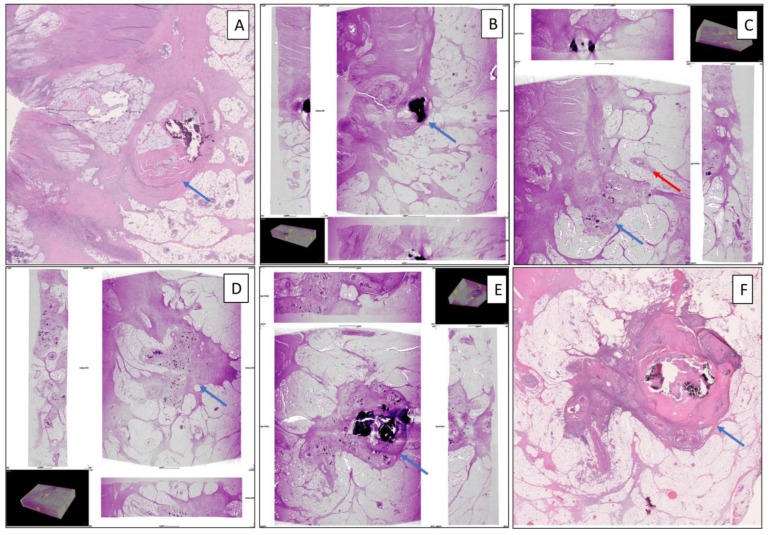
Demonstration of the tumor deposit formation with higher magnification and higher resolution than Figure 2 by conventional hematoxylin and eosin slide image using WSI and micro-CT WBI. (**A**) The relationship of the tumor (blue arrow) and the perirectal adipose tissue on WSI. (**B**–**E**) Micro-CT WBI of the next three consecutive whole-mount blocks shows the relationship between the tumor deposit (blue arrow) and a focus of extramural vascular invasion (red arrow) at a resolution of 9.8–11.7 μm/voxel. (**F**) WSI of the fully formed tumor deposit (blue arrow). (Micro-CT WBI: whole-block imaging using micro-computed tomography; WSI: whole-slide imaging).

WBI detected viable carcinoma in 15/15 blocks that contained viable tumor also detected by WSI. Mucin and LNs were detected equally accurately. Six of the eleven TDs detected on WSI were visualized using WBI. The average size of TDs visualized using WBI was 4.4 mm. The remaining five TDs had an average greatest diameter of 0.8 mm, and they were not visualized using WBI. Upon tracking through consecutive blocks of the visible TDs on WBI (clockwise and coded accordingly), all six TDs originated from the main tumor and extended from the main tumor to perirectal adipose tissue (Supplementary Video S1). In addition, normal vessels (without intravascular tumor emboli) were observed near the extramural tumor extension: eventually, the vessels were blocked by two TDs. One focus of extramural venous invasion accompanied the extramural tumor extension in one of the TDs (Figure 3). However, foci of PNI (without EMVI) and intramuscular vascular invasion (IMVI) were not visualized by micro-CT WBI, which might be due to the small sizes of those histologic features and the relatively lower resolution of micro-CT WBI compared to WSI.

## 4. Discussion

This pilot study showed that micro-CT WBI of consecutive blocks in parallel with corresponding WSI can serve as a means to trace the origin and path of individual tumor deposits in colorectal cancer resection specimens. This method also allowed the evaluation of various other pertinent histologic features including viable tumor, mucin, EMVI, and lymph node three-dimensionally. Detailed assessment of our study case revealed that the primary tumor was the sole origin for six of six TDs evaluated, and while one TD was accompanied by EMVI, none originated from vascular tumor emboli.

The utility of micro-CT WBI in evaluating tumor pathology has been demonstrated in previous studies of colon, lung, breast, and thyroid cancers [2,3,11,12,13]. In all these studies, micro-CT WBI allowed the visualization of detailed structures and histological features. Xu et al. detected capsular invasion as satellite tumor nodules beyond the tumor capsule by showing the penetration point using WBI and 3D reconstruction in thyroid carcinoma [3]. Inoue et al. highlighted that tissue patterns, such as lepidic, acinar, papillary, solid, and micropapillary patterns, that spread through air spaces (STAS) in lung cancers could be detected by micro-CT WBI [11]. In the current study, we further demonstrated micro-CT WBI’s utility in assessing rectal cancer specimens. The 3D reconstruction afforded by this technique provided a means to trace the origin and path of multiple TDs. At the same time, it also allowed the visualization of other important histologic features including tumor, mucin, EMVI, and LNs. An additional value of micro-CT WBI was the ability to examine the entire specimen in a consequential manner without exhausting any tissue in the blocks.

The finding that the primary cancer mass was the origin of all TDs studied in our rectal cancer case is of interest. This may reflect tumor cell invasiveness. Indeed, Fan et al. suggested that TDs may be associated with Twist (a transcriptional factor that induces epithelial–mesenchymal transition and inhibits E-cadherin), which increases cellular motility, and hence facilitates tumor invasion via tumor cell migration to adipose tissue [14].

Notably, our pilot study is primarily aimed at addressing the feasibility of utilizing the micro-CT WBI technique to evaluate the origin of TDs; it was not aimed at determining the frequency of each of the possible origins that could give rise to CRC TDs. The current literature suggests that TDs are mostly associated with lymphatics, vessels, or nerves [15], i.e., TDs mostly originate from EMVI, EM-PNI, and lymph node metastasis (when the node is entirely replaced by tumor). However, the existing literature used methodologies (such as level sections and immunostains) that are suboptimal in tracing TDs longitudinally; hence, the results may not optimally reflect the true origin of the TDs [16].

In light of the well-acknowledged potential prognostic value of TDs in CRC, our findings are of significance. We showed that micro-CT WBI can serve to depict the path of TDs, as well as other related histologic features. A limitation of our study is that we evaluated only one case. Future studies of case series utilizing this technique are warranted to help elucidate how CRC TDs form and what their clinical relevance may be.

## Data Availability

The datasets used during the current study are available from the corresponding author on reasonable request.

## Supplementary Materials

The following supporting information can be downloaded at: https://www.mdpi.com/article/10.3390/diagnostics14161704/s1, Video S1: Whole-Block Imaging of 3D reconstruction of four consecutive blocks.
